# Unexpected Delayed Incursion of Highly Pathogenic Avian Influenza H5N1 (Clade 2.3.4.4b) Into the Antarctic Region

**DOI:** 10.1111/irv.70010

**Published:** 2024-10-17

**Authors:** Simeon Lisovski, Anne Günther, Meagan Dewar, David Ainley, Fabián Aldunate, Rodrigo Arce, Grant Ballard, Silke Bauer, Josabel Belliure, Ashley C. Banyard, Thierry Boulinier, Ashley Bennison, Christina Braun, Craig Cary, Paulo Catry, Augustin Clessin, Maelle Connan, Edna Correia, Aidan Cox, Juan Cristina, Megan Elrod, Julia Emerit, Irene Ferreiro, Zoe Fowler, Amandine Gamble, José P. Granadeiro, Joaquin Hurtado, Dennis Jongsomjit, Célia Lesage, Mathilde Lejeune, Amanda Kuepfer, Amélie Lescroël, Amy Li, Ian R. McDonald, Javier Menéndez‐Blázquez, Virginia Morandini, Gonzalo Moratorio, Teresa Militão, Pilar Moreno, Paula Perbolianachis, Jean Pennycook, Maryam Raslan, Scott M. Reid, Roanna Richards‐Babbage, Annie E. Schmidt, Martha Maria Sander, Lucy Smyth, Alvaro Soutullo, Andrew Stanworth, Léo Streith, Jérémy Tornos, Arvind Varsani, Ulrike Herzschuh, Martin Beer, Michelle Wille

**Affiliations:** ^1^ Polar Terrestrial Systems Alfred Wegener Institute Helmholtz Centre for Polar and Marine Research Potsdam Germany; ^2^ Institute of Diagnostic Virology Friedrich‐Loeffler‐Institut Greifswald Germany; ^3^ Future Regions Research Centre Federation University Australia Ballarat Australia; ^4^ H.T. Harvey & Associates Ecological Consultants Los Gatos California USA; ^5^ Laboratorio de Virología Molecular, Centro de Investigaciones Nucleares, Facultad de Ciencias Universidad de la República Montevideo Uruguay; ^6^ Laboratorio de Evolución Experimental de Virus Institut Pasteur de Montevideo Montevideo Uruguay; ^7^ Point Blue Conservation Science Petaluma California USA; ^8^ Dynamic Macroecology Swiss Federal Research Institute for Forest, Snow and Landscape Research WSL Birmensdorf Switzerland; ^9^ Global Change Ecology and Evolution Research Group (GloCEE), Department of Life Sciences University of Alcalá Madrid Spain; ^10^ Department of Virology Animal and Plant Health Agency‐Weybridge Addlestone Surrey UK; ^11^ WOAH/FAO International Reference Laboratory for Avian Influenza, Swine Influenza and Newcastle Disease Virus Animal and Plant Health Agency‐Weybridge Addlestone Surrey UK; ^12^ Centre d'Ecologie Fonctionnelle et Evolutive (CEFE) Université Montpellier Montpellier France; ^13^ British Antarctic Survey Cambridge UK; ^14^ Institute for Ecology and Evolution Friedrich Schiller University Jena Jena Germany; ^15^ Thermophile Research Unit, Te Aka Mātuatua‐School of Science, Te Whare Wānanga o Waikato University of Waikato Hamilton New Zealand; ^16^ MARE–Marine and Environmental Sciences Centre ARNET–Aquatic Research Network Ispa – Instituto Universitário Lisbon Portugal; ^17^ Centro de Estudos do Ambiente e do Mar (CESAM), Departamento de Biologia Animal Faculdade de Ciências da Universidade de Lisboa Lisbon Portugal; ^18^ Department of Public and Ecosystem Health Cornell University Ithaca New York USA; ^19^ Ecole Normale Superieure de Lyon Lyon France; ^20^ Department of Zoology, Marine Apex Predator Research Unit (MAPRU), Institute for Coastal and Marine Research Nelson Mandela University Gqeberha South Africa; ^21^ Falklands Conservation Stanley Falkland Islands; ^22^ Cavanilles Institute of Biodiversity and Evolutionary Biology University of Valencia Valencia Spain; ^23^ Depto. Ecologia Evolutiva, Museo Nacional de Ciencias Naturales CSIC Madrid Spain; ^24^ Departamento de Ecologia y Gestion Ambiental, Centro Universitario Regional del Este Universidad de la Republica Maldonado Uruguay; ^25^ Biodesign Center for Fundamental and Applied Microbiomics, Center for Evolution and Medicine, School of Life Sciences Arizona State University Tempe Arizona USA; ^26^ Institute of Biochemistry and Biology University of Potsdam Potsdam Germany; ^27^ Institute of Environmental Science and Geography University of Potsdam Potsdam Germany; ^28^ Centre for Pathogen Genomics, Department of Microbiology and Immunology, at the Peter Doherty Institute for Infection and Immunity The University of Melbourne Melbourne Victoria Australia; ^29^ WHO Collaborating Centre for Reference and Research on Influenza Peter Doherty Institute for Infection and Immunity Melbourne Victoria Australia

**Keywords:** H5N1, Antarctica, Migratory birds

## Abstract

The current highly pathogenic avian influenza H5N1 panzootic is having substantial impacts on wild birds and marine mammals. Following major and widespread outbreaks in South America, an incursion to Antarctica occurred late in the austral summer of 2023/2024 and was confined to the region of the Antarctic Peninsula. To infer potential underlying processes, we compiled H5N1 surveillance data from Antarctica and sub‐Antarctic Islands prior to the first confirmed cases.

The increasing intensity of highly pathogenic avian influenza virus (HPAIV) H5N1 clade 2.3.4.4b outbreaks have had a substantial impact on poultry and wildlife [1]. Wild bird movements have underpinned the rapid spread of this virus, which has swept across most continents within a 2‐year time span [2]. Compared to previous HPAIV subtypes and clades, some genotypes of H5N1 2.3.4.4b have significantly improved replication in wild birds [3] and increased fitness through continuous reassortments [4], which has likely contributed to a shift in infection dynamics leading to the infection of a broader range of avian species [1]. In addition to their role in viral dissemination, wild birds are suffering huge losses associated with mass mortality events, and the scale of mortality among wild birds being in the millions rather than the tens of thousands reported [5]. Thus, the recent panzootic is a serious conservation concern for a large range of wild bird species.

Due to the absence of waterfowl species that migrate to the Antarctic and sub‐Antarctic islands, the incursion risk of HPAIV in these southernmost regions had been considered low prior to 2021. However, waterfowl are present on islands in northern fringes of the Southern Ocean, and beyond waterfowl, millions of wild birds follow known migration and post‐breeding dispersal routes establishing links and thereby substantial global connectivity. This connectivity includes links to regions with recent HPAIV H5N1 outbreaks involving seabirds and marine mammals [2]. Despite the purported remoteness, low‐pathogenicity avian influenza viruses and antibodies against these viruses have previously been detected in various seabird species nesting at sites along the Antarctic Peninsula and South Shetland Islands, with viral genomes illustrating phylogenetic connectivity to viruses circulating on other continents [6, 7]. As a result, the Scientific Committee on Antarctic Research (SCAR) Antarctic Wildlife Health Network (AWHN) had considered the risk of incursion of the recent panzootic HPAIV H5 into the Antarctic region in the 2022/2023 summer season to be high [8], and considerably higher in 2023/2024 following virus spread to the southernmost regions of South America [9], and confirmed cases in various species including Magellanic penguins ( *Spheniscus magellanicus* ) and Humbold penguins ( *Spheniscus humboldti* ) and several species of marine mammals.

To identify possible incursions of H5N1 into the Antarctic region during the summer season 2022/2023 and the early season 2023/2024, we sampled migratory seabirds at different locations across Antarctica and in sub‐Antarctic areas (Figure 1) and collated a range of observational data. Herein, we define Antarctica as the region south of the Antarctic Polar Front and adjacent islands in sub‐Antarctic areas. In particular, we aimed to collect information pertaining to signs of unusual mortality and known clinical signs of HPAIV infection including loss of coordination and balance, trembling head and body, lethargy, respiratory distress and conjunctivitis [8]. Across all locations, samples were collected in accordance with institutional animal ethics approval, and sample testing was performed with national frameworks (details available in Data S1 Supporting Information).

**FIGURE 1:**
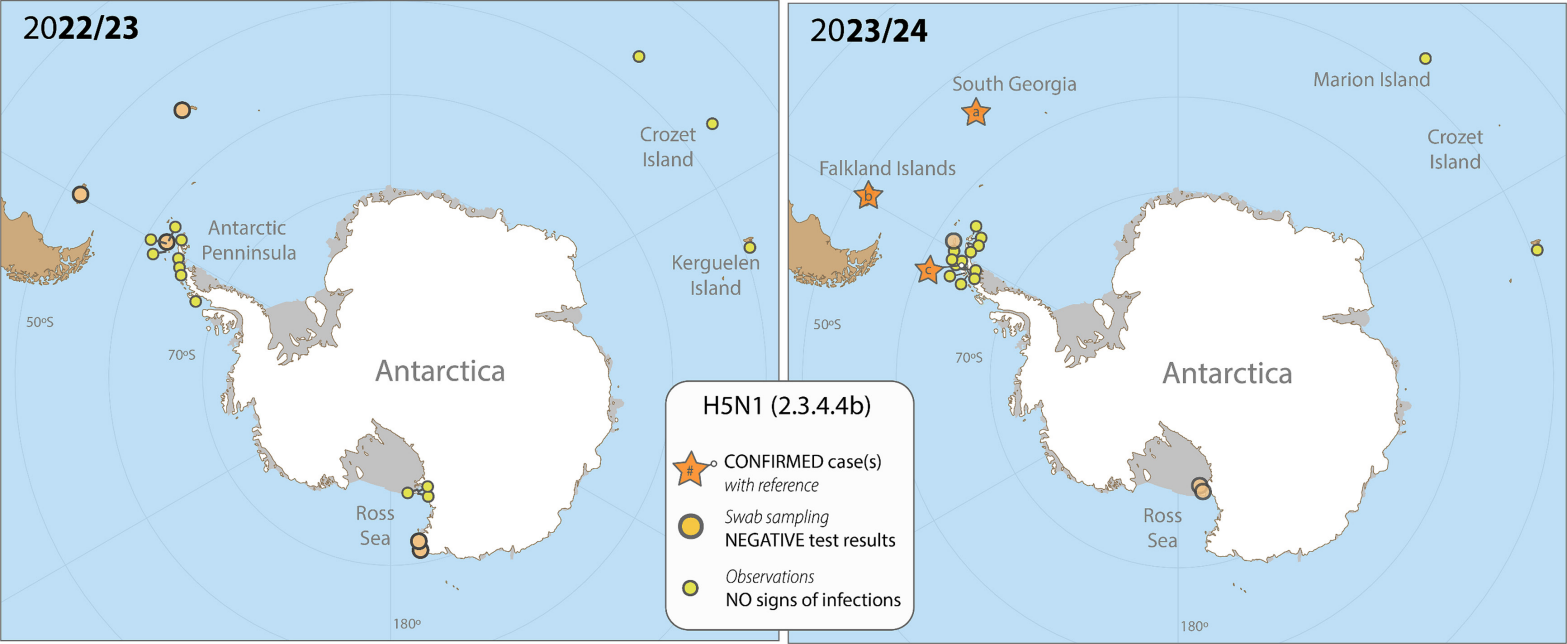
Sampling locations for RT‐qPCR analysis and the detection of H5N1 2.3.4.4b, as well as locations with intensive observational efforts to identify signs of HPAIV infections within breeding bird communities for the breeding season 2022/2023 (left) and 2023/2024 (right). In addition, locations of confirmed cases of infection in 2023/24 (right) are included. Numbers refer to the following references: (a) technical annex, (b) Bennison et al. 10, (c) reported by Antonio Alcami, Angela Vazquez, the PERPANTAR project and researchers from the Instituto Antártico Argentino 12. Maps created with Natural Earth.

Overall, sampling and observational efforts were conducted from early November 2022 to late March 2023 and from October 2023 until the end of February in 2024. Surveillance efforts included a large range of species (i.e., penguins, gulls, skuas and petrels; see technical annex for more information) and locations. In 2022/2023, samples for HPAIV testing were collected from apparently healthy birds from 20 locations in the sub‐Antarctic and Antarctic regions. There were several suspicious observations of dead wild birds on the Falkland Islands (gentoo penguin *Pygoscelis papua* , cattle egret *Bubulcus ibis* ) and South Georgia Islands (wandering albatross *Diomedea exulans* ). However, all swab samples collected from these animals, in addition to swab samples from apparently healthy wild birds in other locations were negative for HPAIV (see technical annex for details on location and species). Together, this strongly suggests that HPAIV H5N1 clade 2.3.4.4b did not enter the Antarctic region during the austral summer 2022/2023 and that the lack of detection was unlikely due to lack of surveillance, testing or disease investigations. This contrasts with the austral summer 2023/24. In October 2023, the first confirmed H5N1 cases were detected in the Falkland Islands and in November on South Georgia Island in the sub‐Antarctic [10, 11] (Figure 1). Given the overlap of species breeding among the Falkland Islands and South Georgia Islands and migrating towards the Antarctic Peninsula and its offshore Islands (e.g., the South Shetland Islands), researchers in the region and the tourist industry have been very diligent in identifying unusual bird behaviour and mortality events. Despite active cases in the Falkland Islands and South Georgia Island, sample collection and observations from 16 locations between November 2023 and early February 2024 in the Antarctic Peninsula and associated islands were negative for HPAIV. Data from the SCAR monitoring project did, however, report suspected cases in the Antarctic region starting in December 2023 [11]. These included brown skuas ( *Stercorarius antarcticus* ) on the South Orkney Islands in December 2023 (no samples collected) and a mortality in brown skuas on Heronia Island in December 2023 (samples collected, HPAIV negative). As of mid‐February, the first positive cases have been reported from the Antarctic Peninsula (reported by Antonio Alcami, Angela Vazquez, the PERPANTAR project and researchers from the Instituto Antártico Argentino, press release [12]). This suggests that H5N1 has spread among colonies in the later breeding season, however, despite the presence of the virus in the region, there has been no evidence thus far for major outbreaks and mass mortality events on the Antarctic Peninsula. Further, based on observation data, the strain did not appear to have reached the Indian Ocean sub‐Antarctic islands as of August 2024 (see Data S1 Supporting Information).

Obviously, incursion risk and successful establishment of HPAIV are contingent on a combination of factors. Most importantly, that host species (i) are infected with HPAIV before travelling into the Antarctic regions, (ii) can migrate long distances despite being infected and (iii) have contact with, and transmit, the virus to susceptible species that could be the starting point of a new epizootic. Most species occupying the Antarctic region are pelagic seabirds with little to no contact with terrestrial birds such as waterfowl, significantly reducing their exposure to outbreaks on land (e.g., South America). However, some species like the brown skua and the giant petrel species (*Macronectes* spp.) are known scavengers (Figure 2), leading to high risks of exposure to HPAIV via the consumption of infected carcasses. It is thus no surprise that brown skuas where among the first confirmed cases both on South Georgia Islands and the Antarctic Peninsula [10]. This species, which can be observed at shorelines of South America, the Falkland Islands and South Georgia Islands [13], may play an important role in spreading the virus. Yet, it seemed that the connectivity established by the seabirds' movements from South America and South Georgia Islands over the Drake Passage to Antarctica is rather limited during the breeding season but might increase again towards the end of breeding activities, when the movement ranges of both adults and first juveniles are extending again. Together with the increasing number of naïve juveniles and concomitant changes in densities, this may explain the delay between initial outbreaks in the Falklands/South Georgia Islands and the first confirmed cases on the Antarctic Peninsula.

**FIGURE 2:**
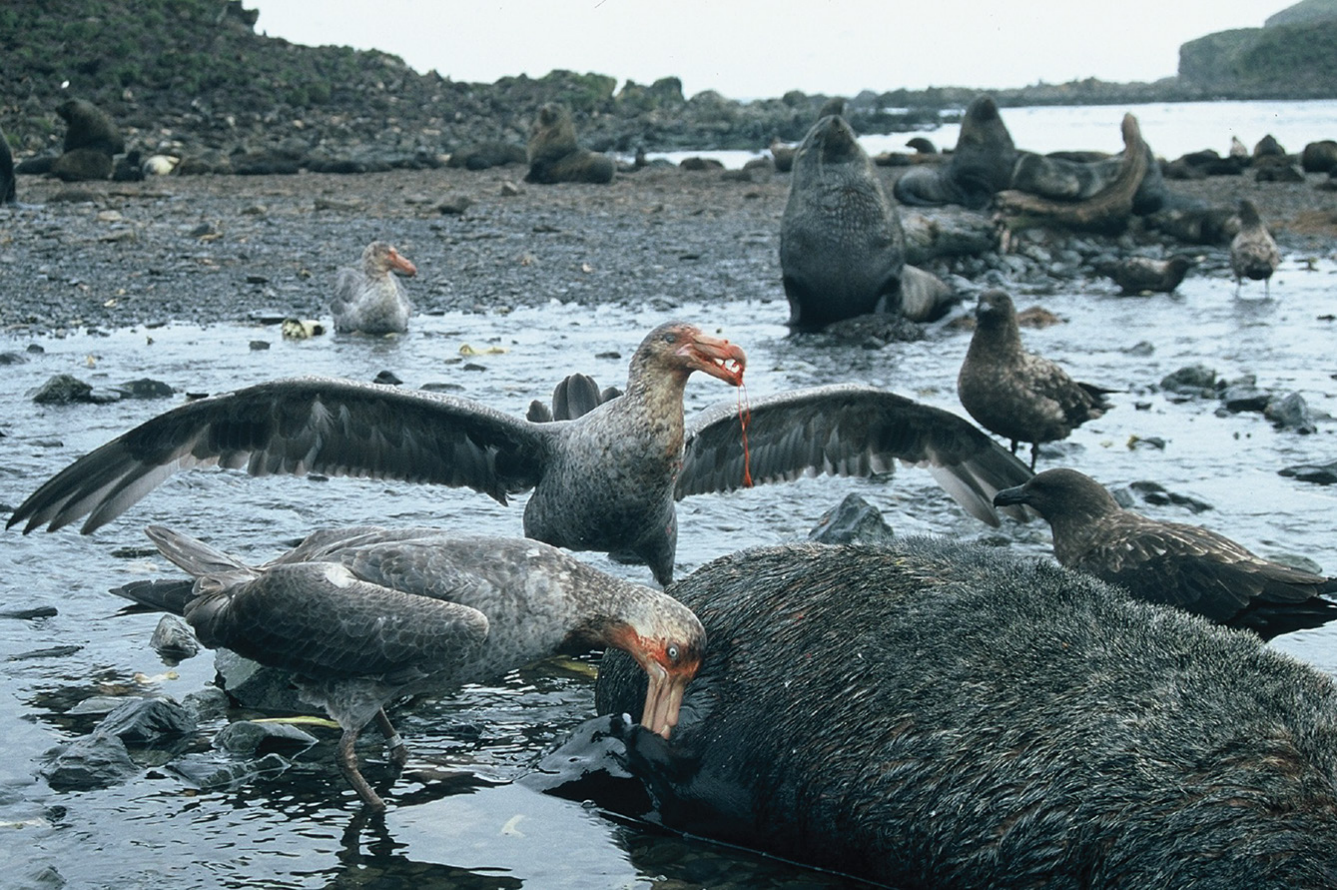
Northern giant petrels and Brown skuas scavenging on an Antarctic fur seal carcass, showing inter‐species interactions with the potential for HPAIV virus transmission. Photo taken on South Georgia by Paulo Catry.

Still, the consequences of viral incursion(s) into Southern Ocean wildlife are unclear, but based on observations from other regions, HPAIV has the potential for devastating effects. Critically, population densities within seabird colonies are often very high, facilitating the transmissions between individuals [14]. Further, prospecting movements of potential recruits, predator–prey interactions and kleptoparasitism between bird species (e.g., skuas, penguins and sheathbills), as well as species scavenging on dead seabirds and mammals, may promote the spread of the virus between colonies [15]. Once the virus has been established in the region, interaction between seabirds and marine mammals may also result in further transmissions, potentially facilitating the adaptation of the virus to mammalian species as suggested in South America [14]. Finally, besides the many seasonal visitors, a sizable portion of animals frequenting the Southern Ocean are endemic to the region and any mass mortality events in Antarctica due to HPAIV H5 are of substantial conservation concern for many species.

Detecting H5N1 incursion(s) and describing the infection dynamics into and within the sub‐Antarctic and Antarctic regions is highly relevant, and standardized surveys for mortality and sampling should therefore be prioritized. These activities should be undertaken with consideration of the potentially zoonotic risks of (emerging) HPAIV H5 [8] and require strict hygiene measures to prevent the spread of the virus through human activities. Sampling and detailed analysis of lineages and virus phenotype will provide crucial information needed to assess risks and respond to future wild bird outbreaks.

## Author Contributions

**Simeon Lisovski:** conceptualization, data curation, formal analysis, visualization, writing – original draft. **Anne Günther:** conceptualization, writing – original draft. **Meagan Dewar:** data curation. **David Ainley:** data curation. **Fabián Aldunate:** data curation. **Rodrigo Arce:** data curation. **Grant Ballard:** data curation. **Silke Bauer:** data curation, project administration. **Josabel Belliure:** data curation. **Ashley C. Banyard:** formal analysis. **Thierry Boulinier:** data curation. **Ashley Bennison:** data curation. **Christina Braun:** data curation. **Craig Cary:** data curation. **Paulo Catry:** data curation. **Augustin Clessin:** data curation. **Maelle Connan:** data curation. **Edna Correia:** data curation. **Aidan Cox:** data curation. **Juan Cristina:** data curation. **Megan Elrod:** data curation. **Julia Emerit:** data curation. **Irene Ferreiro:** data curation. **Zoe Fowler:** data curation. **Amandine Gamble:** data curation. **José P. Granadeiro:** data curation. **Joaquin Hurtado:** data curation. **Dennis Jongsomjit:** data curation. **Célia Lesage:** data curation. **Mathilde Lejeune:** data curation. **Amanda Kuepfer:** data curation. **Amélie Lescroël:** data curation. **Amy Li:** data curation. **Ian R. McDonald:** formal analysis. **Javier Menéndez‐Blázquez:** data curation. **Virginia Morandini:** data curation. **Gonzalo Moratorio:** data curation. **Teresa Militão:** data curation. **Pilar Moreno:** data curation. **Paula Perbolianachis:** data curation. **Jean Pennycook:** data curation. **Maryam Raslan:** data curation. **Scott M. Reid:** data curation. **Roanna Richards‐Babbage:** data curation. **Annie E. Schmidt:** data curation. **Martha Maria Sander:** data curation. **Lucy Smyth:** data curation. **Alvaro Soutullo:** data curation. **Andrew Stanworth:** data curation. **Léo Streith:** data curation. **Jérémy Tornos:** data curation. **Arvind Varsani:** data curation. **Ulrike Herzschuh:** data curation. **Martin Beer:** conceptualization, writing – original draft. **Michelle Wille:** conceptualization, writing – original draft.

## Conflicts of Interest

The authors declare no conflicts of interest.

### Peer Review

The peer review history for this article is available at https://www.webofscience.com/api/gateway/wos/peer‐review/10.1111/irv.70010.

## Supporting information


**Data S1** Supporting Information.

## Data Availability

The data that support the findings of this study are published in the Supporting Informaion (Data S1 Supporting Information).
